# Advancing total uronic acid quantification using a stable isotope dilution approach: validation and application to plant- and algal-derived polysaccharides

**DOI:** 10.1007/s00216-026-06649-1

**Published:** 2026-07-02

**Authors:** Johanna Braun, Maren Punke, Mirko Bunzel

**Affiliations:** Institute of Applied Biosciences, Department of Food Chemistry and Phytochemistry, Karlsruhe Institute of Technology, 76327 Karlsruhe, Germany

**Keywords:** Stable isotope dilution analysis, LC-orbitrap-MS, Polysaccharides, Uronic acids, Algae, Cell wall

## Abstract

Uronic acids (UAs) are key components of plant and algal polysaccharides and serve as crucial markers for their structural and functional characterization. The widely applied conventional colorimetric Blumenkrantz and Asboe-Hansen assay suffers from matrix-dependent interferences and poor reproducibility. In this study, an LC–MS-based stable isotope dilution approach was optimized for reliable quantification of total UA content in complex biopolymer matrices. Calibration and degradation parameters were systematically adapted to various UA compositions, including galacturonic, glucuronic (GlcA), mannuronic (ManA), and guluronic acids (GulA). Structural factors such as degree of polymerization, *O*4-methyl substitution of GlcA, and the stability of aldobiuronic acid linkages in glucuronoxylan were also evaluated. Comparison with spectrophotometric results revealed that the LC–MS method provides higher robustness and selectivity, particularly in samples prone to over- or underestimation by traditional assays. The method further allows selective determination of alginate-derived UAs (ManA + GulA) in mixed matrices, exemplified by food materials containing xanthan. The optimized LC–MS method thus represents a versatile and accurate approach for the quantitative assessment of total UA contents in diverse biological and industrially relevant polysaccharides.

**Graphical abstract:**

**Figure:**
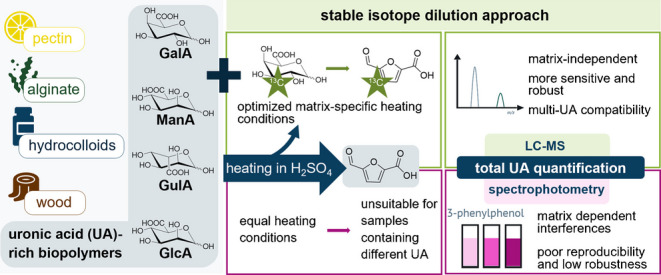

**Supplementary Information:**

The online version contains supplementary material available at 10.1007/s00216-026-06649-1.

## Introduction

Uronic acids (UAs) represent a central class of monosaccharides widely distributed across biological systems. They are integral components of many plant polysaccharides, contributing critically to their structural and functional properties. For instance, pectin features a galacturonic acid (GalA)-rich backbone and serves as a key component of the middle lamella and primary cell walls of dicotyledonous plants [1]. The linear polymer alginate, composed of alternating 1,4-linked β-d-mannuronic (ManA) and α-l-guluronic acid (GulA) residues, is characteristic of brown algal cell walls [2]. In lignocellulosic biomass, glucuronoxylans (GX) are representative hemicelluloses featuring α-d-glucuronic acid (GlcA) substitutions at the *O*2-position of the xylose (xyl) units [3]. Furthermore, hydrocolloids such as xanthan, gum arabic, and karaya gum contain varying proportions of GalA and/or GlcA, which significantly influence their physicochemical behavior [4–6].

These UA-containing polysaccharides play central roles in plant structure and physiology, while also being of great technological and industrial relevance. Often, their pronounced water solubility and gel-forming capabilities make them indispensable as thickeners, stabilizers, and emulsifiers in food systems such as yogurts, dressings, and beverages [7–9]. They are also employed in microencapsulation and controlled-release pharmaceutical formulations, and increasingly in medical applications such as wound healing and bioactive coatings [10].

Given their high relevance and increasing demand in both research and industry, the analysis of UA-containing biopolymers has become a key aspect of carbohydrate analysis. Central to this analytical characterization is the determination of the total UA content, which provides an essential compositional parameter for elucidating the structure and functional behavior of polysaccharides.

**Fig. 1:**
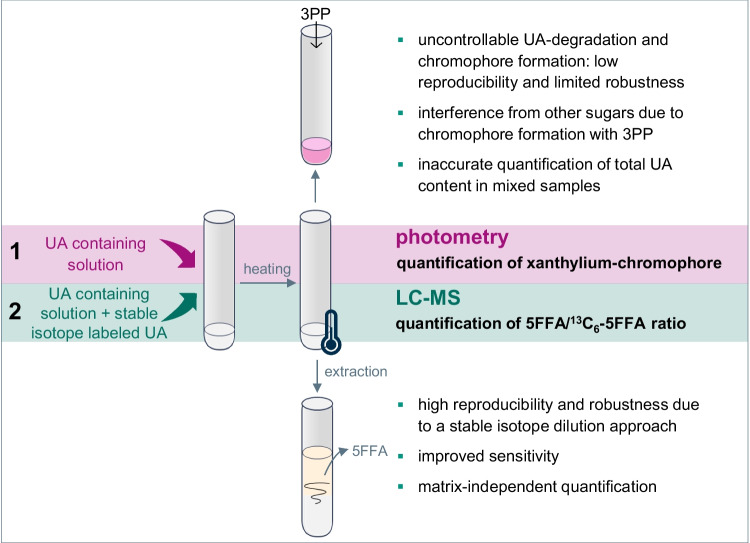
Comparison of colorimetric uronic acid (UA) quantification according to (**1**) according to Blumenkrantz and Asboe-Hansen [14] and LC–MS-based quantification as described by Braun and Bunzel [18] (**2**). Both methods rely on the acid-catalyzed thermal degradation of UA in concentrated sulfuric acid. Photometric detection is based on the formation of a xanthylium chromophore resulting from the reaction of the UA degradation product 5-formyl-2-furancarboxylic acid (5FFA) with 3-phenylphenol (3PP). In contrast, the LC–MS-based stable isotope dilution assay directly quantifies 5FFA, achieving enhanced robustness through the addition of a ^13^C-labeled UA internal standard

**Fig. 2:**
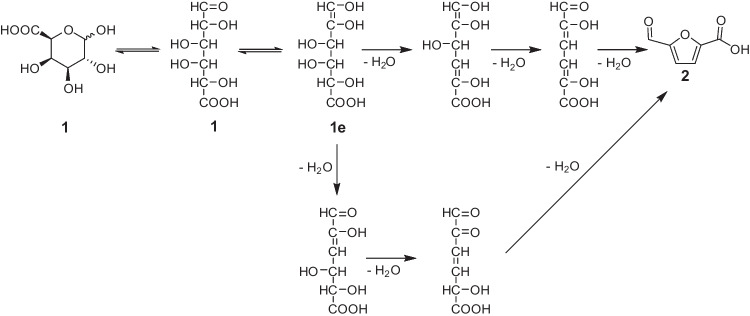
Mechanism of galacturonic acid (1) dehydration. Starting from the 1,2-endiol (1e), unsaturated reactive intermediates are formed through the elimination of water at different positions, leading to 5-formyl-2-furan carboxylic acid (2) [20, 21]

The first attempts to quantify UAs date back to Mann and Tollens [11], who measured the CO₂ released upon decarboxylation as a proxy for UA content. Due to nonspecific reactions with other matrix components, this approach proved unsuitable [12, 13]. Subsequently, colorimetric assays became established, notably the method developed by Blumenkrantz and Asboe-Hansen [14], which remains the most widely used procedure for total UA determination, with more than 6500 citations to date. This assay involves the degradation of UAs in concentrated sulfuric acid, followed by the reaction of the resulting products with 3-phenylphenol (3PP) to form an intensely magenta-colored chromophore (Fig. 1 (1)). Braun et al. [15] identified this chromophore as the (5-carboxyfuran-2-yl)−3,6-diphenyl-9*H*-xanthen-9-ylium ion, formed via condensation of two 3PP molecules with 5-formyl-2-furancarboxylic acid (5FFA), a characteristic degradation product of UAs in concentrated acid (Fig. 2) [16]. However, other furan derivatives arising from the degradation of neutral sugars may also react with 3PP to form interfering chromophores, leading to inaccuracies in quantification. Moreover, variations in the degradation efficiency compromise the robustness and reproducibility of the spectrophotometric method [15, 17].

These limitations are overcome by a liquid chromatography–mass spectrometry (LC–MS)-based approach developed as a stable isotope dilution analysis. With this method, 5FFA formed during the acid degradation of GalA-containing oligo- and polysaccharides is directly quantified after extraction using ^13^C-labeled GalA as internal standard (Fig. 1 (2)). Addition of this internal standard before acid degradation helps to compensate for matrix-dependent variations, analyte losses during analysis, and, most importantly, non-reproducible acid degradation and, thus, inconsistent 5FFA formation. Consequently, this approach enables precise and sensitive quantification of total GalA content in, for example, pectins [18, 19].

While our previous work established the principle of stable isotope dilution LC–MS for GalA-rich pectic matrices [18], its applicability to the full spectrum of biologically and industrially relevant UAs remained unexplored. This study addresses this gap by systematically expanding and adapting this approach to include GlcA, ManA, and GulA, thereby providing the first robust LC–MS-based methodology for total UA quantification in diverse and complex biopolymer matrices such as algae and plant exudates. Furthermore, this work provides a novel, in-depth evaluation of how structural features, including the degree of polymerization (DP), specific substitutions (4-*O*-methylation), and the inherent stability of aldobiuronic acid linkages found in glucuronoxylans (GX), impact the quantification process, addressing critical parameters that have remained largely unexplored in previous methodological reports.

## Material and methods

### Chemicals

d-GlcA, isomaltotriose, xanthan gum, and 2-deoxy-d-glucose were purchased from Merck KGaA (Darmstadt, Germany). d-ManA sodium salt and l-GulA sodium salt were obtained from Biosynth (Staad, Switzerland), and 4-*O*-methyl-d-GlcA (4-*O*-Me-GlcA) was purchased from Santa Cruz Biotechnology (Dallas, TX, USA). d-[UL-^13^C₆]GalA potassium salt (UL: uniformly labeled) and d-[UL-^13^C₆]GlcA sodium salt were obtained from Omicron Biochemicals, Inc. (South Bend, IN, USA). 3PP and 5FFA were purchased from Apollo Scientific Ltd (Manchester, UK). Beechwood xylan was obtained from Neogen (Lansing, MI, USA), and sodium alginate was purchased from AppliChem (Darmstadt, Germany). Gum arabic and karaya gum were purchased from Thermo Scientific (Waltham, MA, USA). Deuterium oxide (D₂O) was obtained from Deutero GmbH (Kastellaun, Germany).

*Endo*-β−1,4-d-xylanases (GH11, EC 3.2.1.8, from *N. patriciarum*; GH10, EC 3.2.1.8, from *C. thermocellum*) were obtained from Megazyme (Bray, Ireland), and from NZYtech (Lisbon, Portugal). The enzyme used for preparative fiber isolation was the protease Alcalase 2.4 L (EC 3.4.23.62 from *B. licheniformis*), obtained from Merck KGaA (Darmstadt, Germany).

All other chemicals and reagents were of analytical grade and purchased from VWR International GmbH (Darmstadt, Germany), Carl Roth GmbH + Co. KG (Karlsruhe, Germany), or Merck KGaA (Darmstadt, Germany).

### Materials and samples

Powdered UA-containing hydrocolloids, namely gum arabic and karaya gum, were purchased from ThermoFisher Scientific (Waltham, MA, USA), and xanthan gum was obtained from Merck KGaA (Darmstadt, Germany). Beechwood and eucalyptus wood were dried, milled, and sieved to obtain a particle size below 0.5 mm. Ground and dried kelp algae (*Ascophyllum nodosum*) and bubble tea pearls were obtained from commercial sources.

Insoluble and soluble fiber (IF and SF) fractions were isolated from the algal material as described in “Preparative fiber isolation”. The bubble tea pearls were freeze-dried, dissolved in warm water under continuous stirring, and subsequently the polysaccharides were precipitated and washed according to the procedure applied for SF isolation (“Preparative fiber isolation”).

### Preparative fiber isolation

IF and SF were prepared from algal samples according to Keller et al. [22] with slight variations. Dried ground material (15 g) was suspended in 400 mL of 0.08 M sodium phosphate buffer (pH 6.2). The pH was set at 7.5 using 0.275 M NaOH, followed by addition of 600 μL protease and incubation at 60 °C for 30 min with agitation. IF was separated by centrifugation and sequentially washed with water, 96% ethanol, and acetone. The supernatant and washings were combined, and SF was precipitated by adding four volumes of 96% ethanol and storing overnight. The SF precipitate was recovered by centrifugation, washed with 80% and 96% ethanol followed by acetone, and dried at 60 °C.

### Monosaccharide composition

SF and IF, as well as the water-soluble hydrocolloids (gum arabic, xanthan, and karaya gum) were hydrolyzed by combined methanolysis/trifluoroacetic acid hydrolysis using 1.25 M HCl in methanol at 80 °C for 16 h, followed by hydrolysis with 2 M trifluoroacetic acid at 121 °C for 1 h [23]. After evaporation, the obtained monosaccharides were dissolved in water and analyzed. IF and wood samples were hydrolyzed using H_2_SO_4_ following Saeman’s principle [24]. Samples were pretreated with 12 M H_2_SO_4_ under ice cooling for 10 min, followed by incubation at room temperature for 2 h. The acid concentration was adjusted to 1.6 M, and samples were hydrolyzed at 100 °C for 3 h. After hydrolysis, the samples were membrane filtered (PTFE syringe filter, 0.45 µm) and neutralized with 4 M NaOH. 2-Deoxy-d-glucose was added as an internal standard for quantification.

Monosaccharide analysis was performed by high performance anion exchange chromatography coupled with a pulsed amperometric detector (HPAEC-PAD, Alexys-HPLC-System with a DECADE Elite detector, Antec Scientific, Hoorn, NL) using a SweetSept AEX200 column (5 µm, 4 mm × 200 mm, Antec Scientific, Hoorn, NL) as previously described by Braun and Bunzel [18]. Results are shown as supplementary data (Figs. S1–S3). 

### Isolation of uronic acid-containing oligosaccharides

#### Alginate oligosaccharides

##### Acid degradation of alginate 

Alginate oligosaccharides (AOS) were prepared following a modified procedure based on Jonathan, Bosch [25]. Sodium alginate (0.5 g) was dissolved in 100 mL of deionized water. The pH was adjusted to 4.0 using 0.25 M hydrochloric acid. Two separate hydrolysis reactions were carried out by incubating the sealed solutions at 100 °C for 2 h and 3 h, respectively. After cooling to room temperature, reaction mixtures were combined, neutralized with 0.275 M sodium hydroxide, and residual polysaccharide was precipitated by the addition of 660 mL of 96% ethanol. The precipitate was removed by centrifugation (7 min, 2683 g), and the solution, containing the AOS, was evaporated. Residual water was eliminated by freeze-drying overnight.

The dried residue was dissolved in 50% acetonitrile (ACN) and directly subjected to semi-preparative HPLC with evaporative light scattering detection (ELSD) for fractionation of alginate oligosaccharides. For product identification and purity evaluation Ultra-High Performance Liquid Chromatography (UHPLC)-MS (“Characterization and semi-preparative isolation”) was used.

##### Characterization and semi-preparative isolation 

The diluted hydrolysate containing the AOS was fractionated by using a semi-preparative HPLC-System (Azura, KNAUER, Berlin, Germany), equipped with a XBridge BEH Amide column (5 µm, 10 mm × 250 mm; Waters Corporation, Milford, MA, USA). A flow rate of 4 mL/min was used, and the temperature was set at 50 °C. Detection was carried out after the eluent was split 1/20, with the smaller fraction being directed to an ELSD (Sedex 85, ERC GmbH, Riemerling, Germany), whereas the larger part was collected by using a multiposition valve (Azura V2.1S, Knauer, Berlin, Germany). A gradient of 20:80 (v/v) (A) and 80:20 (v/v) (B) of ACN:water each containing 0.2% formic acid was used as follows: starting with 20% A; 0–30 min, linearly to 70% A; 30–55 min linearly to 100% A; 55–60 min, held at 100% A; 60–65 min linearly back to 20% A; equilibration with 20% A for further 10 min. The fractions were evaporated, freeze-dried, and weighed.

In accordance with Jonathan, Bosch [25] hydrophilic interaction liquid chromatography (HILIC) was employed to identify the oligosaccharides in the hydrolysate and the fractions, as well as to calculate the purity of the corresponding AOS. The analysis was carried out on a Nexera X2 UHPLC System (Shimadzu, Kyõto, Japan) coupled with an ESI–MS detector (LCMS 2020, Shimadzu, Kyõto, Japan). Chromatographic separation was performed on an Acquity UPLC BEH Amide column (1.7 µm, 2.1 mm × 150 mm) in combination with a VanGuard precolumn (1.7 µm, 2.1 mm × 5 mm; Waters Corporation, Milford, MA, USA). Elution was performed at a flow rate of 0.4 mL/min at 50 °C. The injection volume was 10 µL. The mobile phase consisted of 20:80 (v/v) (A) and 80:20 (v/v) (B) of ACN:water, each containing 0.2% formic acid and 10 mM ammonium formate. The following elution gradient was applied: 0–1 min, held at 0% A, 1–60 min, linearly to 60% A; 60–65 min, linearly to 100% A; held for 5 min; 70–75 min, linearly back to 0% A; equilibration for further 5 min with 0% A. ESI–MS detection was performed using the negative ionization mode and selected ion monitoring (SIM) (−4.5 kV) at a nebulization gas flow (nitrogen) of 1.5 L/min and 15 L/min drying gas. The interface temperature was set to 350 °C, the desolvation line temperature was 250 °C, and the heating block was operated at 200 °C. All AOS (DP 1 to 15) were detected simultaneously by monitoring their [M-H]^−^ ions. For oligosaccharides with DP6 and higher (up to DP 15), [M-2H]^−^ ions were included. The same molar detector response was assumed for all AOS (see Table S1).

#### (Glucurono)xylan oligosaccharides

##### Enzymatic hydrolysis of beechwood xylan

To optimize enzymatic digestion, 1.5 mL of a beechwood xylan standard solution (10 mg/mL) was incubated with varying amounts of GH10 or GH11 xylanases (0.5 µL, 1 µL, 2 µL, and 5 µL). Incubations were performed at 75 °C for GH10 or 50 °C for GH11, for time periods ranging from 1.5 h to 24 h. Enzyme activity was terminated by adding ethanol to a final volume of 2 mL, followed by centrifugation for 5 min. An aliquot of the supernatant (400 µL) was evaporated and reconstituted in 500 µL of water. Samples were diluted appropriately and spiked with 10 µM isomaltotriose before analysis by HPAEC-PAD/MS (“HPAEC-PAD/MS analysis of (glucurono)xylan oligosaccharides”).

##### HPAEC-PAD/MS analysis of (glucurono)xylan oligosaccharides 

Oligosaccharides were analyzed using a Dionex ICS-6000 system (ThermoFisher Scientific, Waltham, MA, USA) equipped with an analytical CarboPac PA200 column (250 × 3.0 mm, 6.0 µm, Waltham, MA, USA). The eluent was split in a 1:2 ratio, with the smaller fraction directed to the PAD and the larger fraction to MS. Prior to MS detection, the eluent was desalted using an electrolytically regenerated suppressor (AERS 500, ThermoFisher Scientific, Waltham, MA, USA). After desalting, a 50 μM lithium chloride solution was added at 0.05 mL/min (AXP-MS pump, ThermoFisher Scientific, Waltham, MA, USA) to facilitate the formation of characteristic lithium adducts for improved sensitivity.

The HPAEC gradient consisted of three eluents: A, water; B, 100 mM NaOH; and C, 100 mM NaOH with 500 mM sodium acetate. Before injection, the column was washed with 100% C (10 min) and equilibrated with 90% A and 10% B (20 min). After injection, the following gradient was applied: 0–10 min, from 90% A and 10% B linearly to 35% A and 65% B; 10–18 min, A kept at 35%, B back to 63.5%, and C linearly to 1.5%; held for 4 min; 11–50 min, A kept at 35%, B linearly to 53%, and C to 12%; held for 7 min; and 57–65 min, isocratic with 100% C.

The flow rate was 0.4 mL/min, with an injection volume of 25 µL and a temperature of 25 °C. Detection was carried out via PAD using a gold working electrode and a PdH reference electrode. MS was operated in positive ion mode, detecting lithium adducts ([M+Li]^+^) of target oligosaccharides in SIM mode, with *m/z* values corresponding to specific xylooligosaccharides (XOS) and glucuronoxylan oligosaccharides (GXOS) (see Table S2).

##### Semi-preparative isolation of (glucurono)xylan oligosaccharides 

For upscaled hydrolysis, 100 mL of beechwood xylan solution (10 mg/mL) was treated with 250 µL of GH11 xylanase. The mixture was incubated at 50 °C for 5 h, followed by enzyme inactivation by the addition of 400 mL ethanol. After centrifugation (10 min, 2683 g), supernatants were pooled and evaporated before freeze-drying.

GXOS were separated from XOS using anion exchange solid-phase extraction (SPE, ISOLUTE® SAX, Biotage, Uppsala, Sweden). Cartridges were conditioned with methanol and equilibrated with water before sample application (1 mL of a 10 mg/mL GXOS-XOS mixture). The cartridges were washed twice with 2 mL of water, and GXOS were eluted with 9 mL of 100 mM formic acid. The eluate was neutralized to pH 5 with 1 M NaOH and freeze-dried. Product composition was monitored by HPAEC as described in “HPAEC-PAD/MS analysis of (glucurono)xylan oligosaccharides”.

Freeze-dried GXOS (90 mg/mL in 25% ACN) were fractionated by using the semi-preparative HPLC-system described in “Characterization and semi-preparative isolation” with a XBridge BEH Amide column (10 × 250 mm, 5 µm, Waters) at 50 °C, and a flow rate of 4 mL/min. A gradient of 20:80 (v/v) (A) and 95:5 (v/v) (B) ACN:water each containing 0.2% formic acid was used as follows: 0–5 min, isocratic with 30% A; 5–35 min, linearly to 51% A; 35–38 min linearly to 90% A; 38–48 min held at 90% A; 48–51 min linearly back to 30% A, equilibration with 30% A for further 10 min. The isolated fractions were evaporated, freeze-dried, and weighed.

##### Characterization by NMR-spectroscopy 

For NMR analysis, 2–3 mg of each GXOS fraction was dissolved in 200 µL of D_2_O and freeze-dried. This procedure was repeated. The dry residue was reconstituted in 500 µL of D_2_O with 0.5 µL of acetone as reference standard (δ_H_ = 2.22 ppm/δ_C_ = 30.89 ppm). 1D and 2D NMR experiments were performed on an Ascend 500 MHz NMR spectrometer (Bruker Biospin, Ettlingen, Germany) equipped with a Prodigy cryoprobe. Standard Bruker pulse sequences and nonuniform sampling (50%) were used.

### Optimization of the degradation of uronic acids to 5FFA

The degradation of UA during heating in concentrated sulfuric acid was optimized (maximum 5FFA concentrations) using a Design of Experiment (DoE, full factorial design) approach with the factors temperature and time following Braun and Bunzel [18]. For each experiment, 1.2 mL of 12.5 mM sodium tetraborate in concentrated H_2_SO_4_ was added to 200 µL of a 55 mg/L aqueous UA solution. The mixture was cooled on ice for 3 min, then incubated at room temperature for 5 min. Samples were heated in a water bath at 60 °C, 77.5 °C, or 95 °C for 5 min, 10 min, or 15 min, followed by cooling on ice for 5 min. After cooling down, the acid was diluted with 0.5 mL of water and 3 mL of 4 M NaOH. Extraction was performed with ethyl acetate (2 × 3 mL), and organic extracts were washed with saturated NaCl solution (2 × 1.5 ml) and dried under a stream of nitrogen. Residues were reconstituted in ACN, which did not dissolve the residual NaCl. ACN solutions were transferred to a new container, dried again and dissolved in 5% aqueous ACN for analysis. All experiments were performed in duplicate.

Quantification of 5FFA was performed on a Nexera LC-40D XS UHPLC System (Shimadzu, Kyōto, Japan) equipped with a UV/Vis detector set at 280 nm. Samples were applied onto a Luna Omega Polar C18 column (150 × 2.1 mm, particle size 1.6 µm; Phenomenex, Torrance, CA, USA) in combination with a polar C18 precolumn (Phenomenex, Torrance, CA, USA) using water containing 0.2% formic acid (A) and ACN (B) as eluents. The gradient and the quantification procedure followed the method of Braun and Bunzel [18].

### Pre-treatment of insoluble material

Insoluble materials were subjected to pre-hydrolysis prior to UA quantification. 5 mg of the materials were treated with 200 µL of 12 M H_2_SO_4_; the suspensions were kept on ice for 15 min and then shaken at room temperature for 2 h. Subsequently, 1.3 mL of water was added, and shaking continued for an additional 10 min. The samples were diluted with 3.5 mL of water and filtered through a 0.45 µm PTFE syringe filter.

### Quantification of total uronic acid content

#### Colorimetric assay

Based on the method described by Blumenkrantz and Asboe-Hansen [14], diluted samples or pretreated insoluble materials (see “Pre-treatment of insoluble material”) were incubated for 5 min at 95 °C in a sodium tetraborate containing concentrated H_2_SO_4_ (12.5 mM). After cooling, the reaction mixture was placed on ice for 5 min, and 3PP (0.15% in 0.5% aqueous NaOH) was added. The absorbance was recorded at 520 nm after a 20 min reaction period. Calibration (10–85 mg/L; except ManA: 25–100 mg/L) was carried out using the UA predominant in each sample, following the recommendation of Yapo [13]. Validation parameters were established as described in “Method validation”.

#### Stable isotope dilution approach using LC–MS

The quantification of total UA contents by LC–MS followed the stable isotope dilution approach developed by Braun and Bunzel [18] for pectic samples. Insoluble material was pretreated as described in “Pre-treatment of insoluble material”. Samples were diluted with water to appropriate concentrations for analysis. Sodium tetraborate in concentrated H_2_SO_4_ (1.2 mL, 12.5 mM) was added to 100 µL of an aqueous, suitably diluted UA-containing solution, along with 100 µL of an aqueous d-[UL-^13^C_6_]GalA (for the analysis of ManA, GulA, ManA + GulA, and GlcA + GalA) or d-[UL-^13^C_6_]GlcA (for GlcA) solution (^13^C_6_-UA; 40 mg/L). The mixture was cooled on ice for 3 min and left at room temperature for 5 min. Heating was performed according to the optimized conditions (“Optimization of the degradation of uronic acids to 5FFA”). After cooling on ice for 5 min, the extraction was carried out as described in “Optimization of the degradation of uronic acids to 5FFA” using ethyl acetate (2 × 3 mL). The final extract was dissolved in 200 µL of 5% aqueous ACN for analysis.

The UA content was quantified via the determination of 5FFA using a Vanquish Flex Binary UHPLC system (ThermoFisher Scientific, Waltham, MA, USA) coupled to a Q Exactive™ Plus Orbitrap-MS detector (ThermoFisher Scientific, Waltham, MA, USA). Chromatographic separation was achieved using a Luna Omega Polar C18 column (150 × 2.1 mm, particle size 1.6 µm; Phenomenex, Torrance, CA, USA) in combination with a polar C18 precolumn (Phenomenex, Torrance, CA, USA). Water containing 0.2% formic acid (A) and ACN (B) were used as eluents. The gradient follows the method of Braun and Bunzel [18]. ESI–MS detection was performed in negative SIM mode with the following parameters: spray voltage of −2.5 kV and capillary temperature of 320 °C. Quantification was achieved by monitoring the [M-H]^−^ ions of 5FFA (*m/z* 139.004) and ^13^C_6_-labeled 5FFA (*m/z* 145.024). The relative peak areas of 5FFA to ^13^C_6_-labeled 5FFA were used for quantification. Calibration was conducted using the corresponding UA-standards (10–85 mg/L, except ManA + GulA: 25–100 mg/L), which were processed and analyzed under identical conditions as the samples.

#### Method validation

Method validation data were processed using *OriginPro 2024* (OriginLab Corporation, Northampton, MA, USA). Calibration curves were prepared in triplicate as outlined in “Colorimetric assay” and “Stable isotope dilution approach using LC–MS”, and mean values were calculated for each concentration level. The normality of data distribution was verified by a Shapiro–Wilk test (α = 0.05) using ten replicate injections of the mid-range calibration point. Homogeneity of variance between sample groups was assessed by an F-test (α = 0.05) based on six replicate injections of the highest and lowest calibration levels [26].

Instrumental repeatability was evaluated from ten replicate injections of the same sample solution, thereby excluding variations from sample preparation. Procedural repeatability was determined from six replicate analyses of both the highest and lowest calibration points. Coefficients of variation (CV, %) were calculated as the ratio of standard deviation to the mean value, and the overall procedural CV was expressed as the arithmetic mean of the two calibration levels.

External recovery was assessed using the corresponding UA standard solution. Matrix effects were determined by spiking pre-analyzed carrot samples with a defined amount of UA.

Limits of detection (LOD) and quantification (LOQ) were determined according to DIN 32645. For this purpose, duplicate calibrations were performed in a concentration range of 2–17 mg/L (GlcA, GalA) or 4–34 mg/L (ManA, GulA), adjusted to include the expected LOQ. LOD and LOQ were calculated applying a critical factor *k* = 3. Inter-day precision was determined in quadruplicate for selected sample materials on two separate days using both LC–MS and spectrophotometric methods. One-way ANOVA (α = 0.05) was performed to evaluate the statistical significance of differences between the days.

## Results and discussion

### Adaptation of reaction conditions for different uronic acids

Quantification of total UA in both colorimetric and chromatographic approaches is based on the formation of 5FFA during acid-catalyzed degradation in concentrated sulfuric acid [27]. In conventional spectrophotometric assays, 5FFA is detected via a secondary color reaction, which can be affected by interfering compounds [13–15, 17]. The stable isotope dilution LC–MS method overcomes this limitation by quantifying 5FFA directly, thereby eliminating the color reaction step and minimizing matrix-dependent interferences.

Previous work demonstrated that 5FFA yields differ markedly between individual UA when subjected to identical hydrolysis conditions, resulting in analyte-specific sensitivities [18]. To achieve optimized sensitivity for each UA in the LC–MS method, degradation parameters (temperature and heating duration in concentrated H_2_SO_4_) were optimized. Earlier spectrophotometric studies have shown that the conversion of UA to furan derivatives depends strongly on these parameters: GalA and GlcA are almost completely degraded at temperatures above 80 °C, whereas ManA and GulA exhibit maximum 5FFA formation around 55 °C [17, 28, 29]. Building on the previously optimized degradation conditions for GalA in pectic matrices (77.5 °C, 5 min), the present study extended the evaluation to GlcA, ManA, and GulA, which are representative UA relevant to plant and algal polysaccharides.

**Fig. 3:**
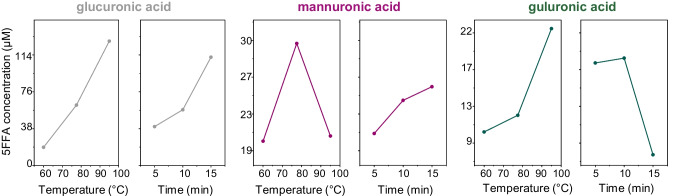
Main effects plots for the degradation of different uronic acids in sodium tetraborate containing concentrated sulfuric acid (12.5 mM). Temperature and reaction time were varied and optimized to achieve maximal formation of 5-formyl-2-furancarboxylic acid (5FFA). Uronic acid concentrations corresponded to the mid-range calibration level (55 mg L^−1^, equivalent to 283 µM)

Two optimization strategies were pursued: (i) maximizing the 5FFA yield for each UA individually, and (ii) harmonizing degradation rates within UA pairs typically co-occurring in natural matrices, such as GalA + GlcA in plants and ManA + GulA in brown algae. Optimization was conducted using a DoE approach, allowing systematic evaluation of main and interaction effects [30]. Reaction temperature (60, 77.5, 95 °C) and heating time (5, 10, 15 min) were selected as experimental factors. Main effects plots for the three UA were employed to visualize the independent influence of each parameter on 5FFA yield (Fig. 3). Steeper slopes indicated stronger factor effects, and curve maxima defined optimal parameter combinations.

As shown in Fig. 3, temperature consistently exerted a greater influence than heating time, suggesting that 5FFA formation in concentrated H₂SO₄ is primarily governed by thermal effects. The response profiles varied among UA, reflecting distinct degradation kinetics. GulA and GlcA displayed stronger time dependencies than ManA, while for ManA and GulA a decline in 5FFA yield at elevated temperatures or time indicated secondary degradation or condensation reactions outpacing 5FFA formation. The optimized degradation parameters derived from these plots are summarized in Table 1.

**Table 1:** Optimized heating times and temperatures for individual uronic acids and their combinations, along with the resulting degradation yields (*5FFA*, 5-formyl-2-furancarboxylic acid; *GalA*, galacturonic acid; *GlcA*, glucuronic acid; *ManA*, mannuronic acid; *GulA*, guluronic acid)

Uronic acid	Temperature (°C)	Time (min)	Uronic acid converted to 5FFA (%)
GalA^a^	77.5	5	33
GlcA	95	15	84
ManA	77.5	15	11
GulA	95	10	10
GalA + GlcA	77.5	15	30
ManA + GulA	60	15	9

^a^Data extracted from Braun and Bunzel [18]

**Fig. 4:**
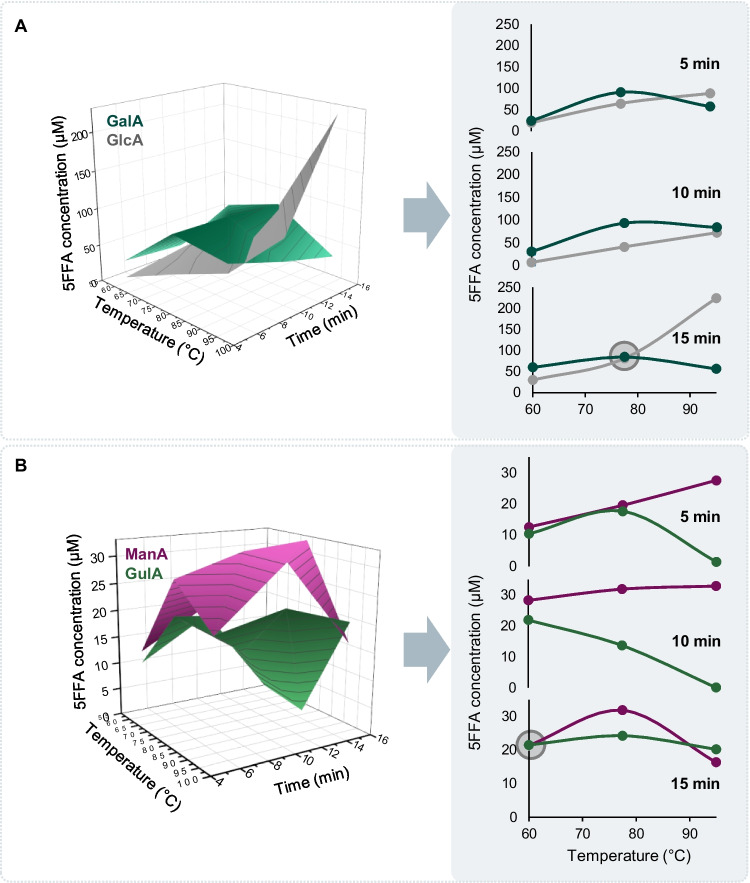
Response surface models illustrating the adaptation of reaction time and temperature in concentrated sulfuric acid to achieve balanced degradation of the uronic acid pairs galacturonic acid (GalA) and glucuronic acid (GlcA) (A) as well as mannuronic acid (ManA) and guluronic acid (GulA) (B) into 5-formyl-2-furancarboxylic acid (5FFA)

These conditions are valid primarily for samples dominated by a single UA. For complex matrices containing multiple UA, degradation rates were balanced to achieve comparable 5FFA yields from equimolar UA concentrations. Based on the DoE results, three-dimensional response surfaces (time × temperature × 5FFA concentration) were generated for GalA + GlcA (Fig. 4A) and ManA + GulA (Fig. 4B). The intersection points of these surfaces identified the reaction conditions producing equivalent 5FFA concentrations for both UA. Graphical analysis revealed intersections at 77.5 °C and 15 min for GalA + GlcA, and at 60 °C and 15 min for ManA + GulA (Table 1). These conditions were subsequently applied to the analysis of plant- and algae-derived samples for the method described in “Stable isotope dilution approach using LC–MS”.

**Fig. 5:**
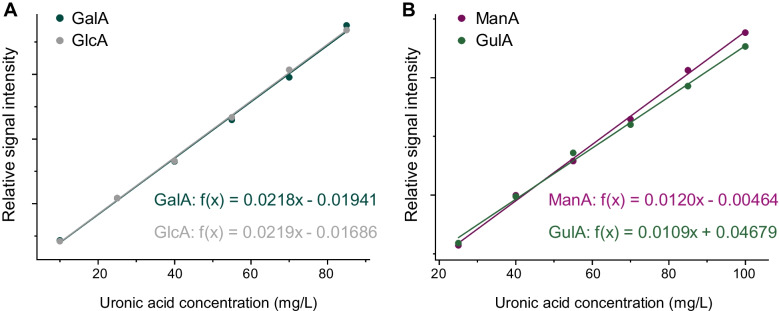
Calibration curves and corresponding regression functions for the uronic acid pairs galacturonic acid (GalA) and glucuronic acid (GlcA) (**A**), and mannuronic acid (ManA) and guluronic acid (GulA) (**B**), obtained under optimized reaction conditions and analyzed by LC–MS. Signals intensities were calculated relative to ^13^C_6_-labeled 5FFA derived from d-[UL-^13^C_6_]GalA

Calibration curves obtained under the optimized conditions for the combined determination of GalA + GlcA (Fig. 5A) and ManA + GulA (Fig. 5B) demonstrated good to excellent agreement. In particular, the calibration functions for GalA and GlcA were almost identical, indicating that comparable UA concentrations within this calibration range result in similar relative peak areas (relative to ^13^C_6_-labeled 5FFA derived from d-[UL-^13^C_6_]GalA) and thus equivalent calculated concentrations. Due to the lower detection sensitivity observed for ManA and GulA, the calibration range was adjusted to 25–100 mg/L. Although the two calibration curves did not overlap completely, their close similarity suggests that no significant deviations in the quantified UA concentrations are expected, regardless of which of the two UA is used for calibration.

Complete conversion of UA to 5FFA was not achieved under any tested conditions (Table 1). The highest recovery (84%) was obtained for GlcA, whereas ManA and GulA yielded only about 10% of the initial substrate as measurable 5FFA. In mixed UA determinations, recoveries decreased further, reflecting the compromise required to balance degradation kinetics between analytes.

### Method validation

The LC–MS method was validated for individually optimized UAs and UA pairs as described in “Method validation”, applying the respective monosaccharide standards and reaction conditions summarized in Table 1. For the pair GalA + GlcA, GalA was used as calibration standard, and the pair ManA + GulA was calibrated against ManA. Although samples containing pure ManA or GulA are mainly of bacterial origin, these UAs were included, too. For comparison, spectrophotometric analyses were performed under the conditions reported by Blumenkrantz and Asboe-Hansen [14] (95 °C, 5 min) according to “Colorimetric assay”. The corresponding validation parameters are summarized in Table 2 (individual UAs) and Table 3 (UA pairs). To ensure accurate quantification, both ^13^C-GalA and ^13^C-GlcA were employed as internal standards. ^13^C-GalA was found to be a suitable internal standard for ManA and GulA because their degradation kinetics to form 5FFA are comparable under the optimized conditions for ManA/GulA, ensuring high measurement accuracy across these different UA species.

**Table 2:** Validation parameters of the LC–MS based quantification of the analyzed uronic acids, that is, glucuronic acid (GlcA), mannuronic acid (ManA), and guluronic acid (GulA) in comparison with the spectrophotometric quantification (*CV*, coefficient of variation; *LOD*, limit of detection; *LOQ*, limit of quantification; *R*^*2*^, coefficient of determination)

	**LC–MS**			**Spectrophotometry**		
	GlcA	ManA	GulA	GlcA	ManA	GulA
**Calibration range (mg/L)**	10–85			10–85	25–85	10–85
**Regression equation**	y = 0.02348x + 0.03231	y = 0.00647x + 0.01052	y = 0.02035x- 0.02035	y = 0.00506x + 0.13604	y = 0.00223x + 0.13581	y = 0.00374x + 0.06965
**R**^**2**^	0.9991	0.9998	0.9992	0.9976	0.9686	0.9906
**LOD (mg/L)**	1.83	2.60	2.93	6.30	16.29	9.72
**LOQ (mg/L)**	6.11	8.82	9.83	23.81	60.11	36.16
**Instrumental CV (%)**	0.86			4.26		
**CV of the procedure (%)**	2.17	2.14	1.56	11.20	12.52	9.22
**External recovery (%)**	100.02 ± 0.06	98.36 ± 1.08	98.31 ± 1.06	113.16 ± 1.55	129.99 ± 3.53	80.76 ± 0.64
**Recovery in matrix (%)**	99.09 ± 0.07^a^	98.61 ± 0.96^b^	99.65 ± 1.16^b^	110.53 ± 8.31^a^	43.83 ± 0.19^b^	38.82 ± 3.67^b^

^a^Beechwood after pretreatment according to “Pre-treatment of insoluble material” was used as the matrix

^b^A blank matrix adjusted to the monosaccharide composition of brown algal polysaccharides was used as the matrix

**Table 3:** Validation parameters of the LC–MS-based quantification of the analyzed uronic acids pairs glucuronic acid (GlcA) and galacturonic acid (GalA) as well as mannuronic acid (ManA) and guluronic acid (GulA) in comparison with the spectrophotometric quantification (*CV*, coefficient of variation; *LOD*, limit of detection; *LOQ*, limit of quantification; *R*^*2*^, coefficient of determination)

	**LC–MS**		**Spectrophotometry**	
	GalA + GlcA^a^	ManA + GulA^b^	GalA + GlcA^a^	ManA + GulA^b^
**Calibration range (mg/L)**	10–85	25–100	10–85	25–100
**Regression equation**	y = 0.0218x - 0.01941	y = 0.0120x - 0.00464	Values are based on the predominant uronic acid present (data Table 2)	
**R**^**2**^	0.9986	0.9990		
**LOD (mg/L)**	2.48	2.94		
**LOQ in mg/L**	8.04	10.29		
**Instrumental CV (%)**	0.86			
**CV of the procedure (%)**	2.56	3.94		
**External recovery (%)**	102.12 ± 0.84	98.86 ± 0.42	63.79 ± 4.00	300.44 ± 6.32
**Recovery in matrix (%)**	101.54 ± 0.52^c^	101.76 ± 0.77^d^	137.20 ± 14.54^c^	130.16 ± 6.39^d^

^a^GalA was used for calibration

^b^ManA was used for calibration

^c^Karaya gum was used as matrix

^d^SDF of the brown seaweed was used as matrix

Within the investigated ranges, the LC–MS method exhibited excellent linearity with correlation coefficients (R^2^) exceeding 0.999, whereas the spectrophotometric assay yielded R^2^ values above 0.96. Based on the slopes of the calibration functions, the LC–MS analysis showed approximately four-fold higher sensitivity than the colorimetric method. LOD for the optimized LC–MS method ranged between 1.83 mg/L (GlcA) and 2.93 mg/L (ManA), with LOQ being approximately three times higher. For UA pairs, which were not optimized for maximum sensitivity but for similar response (5-FFA formation), the respective limits were slightly elevated as expected.

Spectrophotometric LODs were on average four times higher. In particular, ManA was only detectable above 16.3 mg/L. This required a calibration range (for quantification) starting at 25 mg/L, which deviated from the original literature protocol. In general, all spectrophotometric calibration curves started below their respective LOQ, restricting data in this range to qualitative interpretation only. This markedly reduces the analytical validity of the spectrophotometric approach, especially for samples with low UA content. Furthermore, it should be noted that while the LC–MS approach does not aim for a significant increase in absolute sensitivity compared to the spectrophotometric assay, it provides a substantial improvement in analytical selectivity.

Instrumental CV were determined from ten replicate measurements of the same sample, reflecting the precision of repeated injections. The LC–MS analysis of 5FFA yielded a mean CV of 0.86% [18], whereas the spectrophotometric determination of the corresponding chromophore from 5FFA and 3PP resulted in a substantially higher value of 4.26%. This difference can be attributed to the lower robustness of the photometric assay, its greater susceptibility to interferences, and its reduced specificity of optical detection.

CV of the procedure, including sample preparation, was determined from six independent analyses. For LC–MS, these ranged between 1.56% and 2.17%, only slightly exceeding the instrumental variation. In contrast, the spectrophotometric assay showed significantly higher values between 9.22% and 12.53%. This indicates greater methodological uncertainty due to the unreproducible decomposition of UA into 5FFA, as well as the color reaction with unknown kinetics [15].

Recovery rates obtained with the LC–MS method were consistently within 100 ± 2%, indicating negligible matrix effects. In contrast, external recoveries determined using the Blumenkrantz–Asboe-Hansen assay were substantially overestimated for most UAs, except GulA. As this overestimation could not be attributed to matrix-derived chromophores, it likely results from non-reproducible degradation or chromophore-formation kinetics, emphasizing the limited reliability of this assay. Even minor deviations in reaction conditions led to disproportionate changes in absorbance, suggesting that the 5FFA chromophore reaction does not proceed stoichiometrically or consistently.

The spectrophotometric assay according to Blumenkrantz and Asboe Hansen proved unsuitable for the simultaneous quantification of multiple UAs within one sample. This was evident from the low external recoveries for the GalA + GlcA mixture and the pronounced overestimation observed for ManA + GulA (Table 3). The discrepancy arises from the different degradation kinetics of individual UAs in concentrated sulfuric acid, which cannot be adequately accounted for within the published conditions of the Blumenkrantz-Asboe-Hansen assay.

### Evaluation of structural influences of the analyzed polysaccharides

In the previous sections, the optimization of the LC–MS method (“Adaptation of reaction conditions for different uronic acids”) and the validation (“Method validation”) were conducted using UA-monosaccharide standards. However, in natural matrices, UAs occur covalently bound within polysaccharide structures, where factors such as DP, linkage type, and chemical substitution may influence their degradation behavior and analytical recovery within the LC–MS method. We demonstrated previously that the DP of homogalacturonan oligosaccharides has no measurable impact on the degradation efficiency. Building upon these findings, the present study extends the evaluation to other UA-based oligomers/polymers to examine whether similar relationships hold for alginate-derived oligomers, methyl ethers of GlcA (4-*O*-Me-GlcA) in GX, and UAs involved in aldobiuronic acid-type linkages.

#### Influence of the degree of polymerization of alginate oligosaccharides

**Fig. 6:**
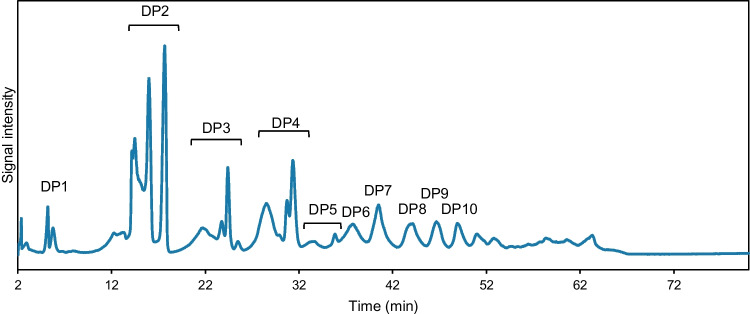
Total ion current (TIC) MS-detected chromatogram that shows the HILIC separation of the final alginate hydrolysate, indicating alginate oligosaccharides of different degrees of polymerization (DP)

To evaluate the effect of the DP, AOS containing ManA and GulA units with DP 3–10 were produced. As no suitable enzyme was available to generate saturated AOS, they were prepared by mild acid hydrolysis of alginate at pH 4. Two hydrolysates differing in hydrolysis time, either 2 h or 3 h, were combined to broaden the oligosaccharide spectrum (Fig. 6). With increasing DP, progressively broader chromatographic signals were observed, reflecting structural heterogeneity resulting from different ManA/GulA ratios and/or sequences. Due to overlapping signals, the chromatographic separation of the different species within each DP was not further pursued as the goal of the experiment was limited to assessing DP effects on analytical performance.

Semi-preparative fractionation yielded AOS of defined DPs, although higher DPs (> 10) could not be isolated in sufficient purity (> 90%). The purified AOS were analyzed using the optimized LC–MS method for ManA + GulA determination (Table 1). Equivalent concentrations (100 µM monosaccharide equivalents) were used for all oligosaccharide and monosaccharide standards, with ManA serving as the 100% recovery reference (Table 4). The recoveries of the analyzed AOS ranged between 95 and 110%. No systematic correlation between DP and recovery was observed. These results indicate that the DP of polyuronic acids exerts no measurable influence on the analytical recovery or degradation efficiency within the examined range. Consequently, the LC–MS method can be applied reliably to both oligomeric and presumably polymeric UA structures.

**Table 4:** Recovery of uronic acids in the isolated alginate oligosaccharides with a defined degree of polymerization (DP), analyzed by the developed LC–MS method with the optimized reaction parameters according to Table 1

DP of the oligosaccharide	Recovery (% monosaccharide)
3	100.45 ± 0.45
4	105.23 ± 0.59
5	97.19 ± 1.45
6	106.45 ± 0.52
7	110.10 ± 3.66
9	108.93 ± 2.55
10	95.27 ± 3.20

#### Influence of methyl ethers of uronic acids

The influence of UA substitution was investigated using the monomer 4-*O-*Me-GlcA, a structural feature characteristic of GX in hardwoods [31]. During degradation in concentrated sulfuric acid, the methoxy group at position *O*4 is released as methanol; however, this substitution may alter the degradation kinetics of GlcA as the methylation prevents intramolecular lactone formation [27, 32]. Thus, Li et al. [32] postulated that 4-*O*-Me-GlcA exhibits a degradation behavior comparable to that of GalA.

**Fig. 7:**
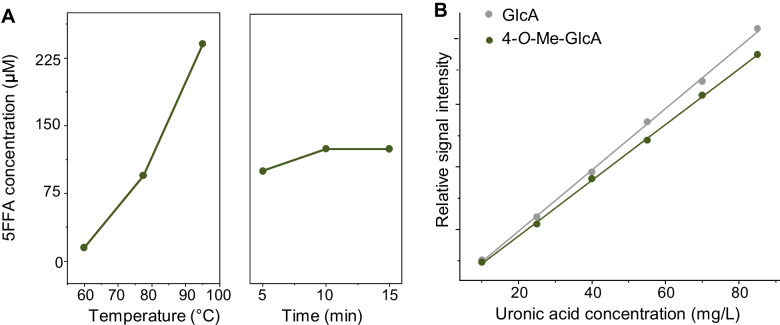
Main effects plot for the degradation of 4-*O*-methyl glucuronic acid (4-*O*-Me-GlcA) to 5-formyl-2-furancarboxylic acid (5FFA) (A), and calibration curves obtained from the LC–MS analysis of glucuronic acid (GlcA) and 4-*O*-Me-GlcA after heating at 95 °C for 15 min (B)

The degradation behavior of 4-*O-*Me-GlcA was evaluated using the DoE approach described in “Adaptation of reaction conditions for different uronic acids”. The resulting main effects plot (Fig. 7A) clearly differed from that of GalA (Fig. 3), contradicting the assumption of identical degradation behavior [32]. The optimal degradation temperature for 4-*O-*Me-GlcA was determined to be 95 °C, with reaction times exerting only a minor effect, comparable to non-methylated GlcA (Fig. 3). Accordingly, the optimized parameters established for GlcA (95 °C, 15 min, Table 1) were also applied to 4-*O*-Me-GlcA-containing solutions.

Calibration curves of GlcA and 4-*O-*Me-GlcA (Fig. 7B) exhibited comparable slopes and linearity, confirming analogous degradation efficiencies. Minor deviations in the upper calibration range result in only slightly higher concentrations when 4-*O-*Me-GlcA-based calibration is used, suggesting that samples containing 4-*O-*Me-GlcA can be principally quantified using the GlcA conditions (Table 1). However, to minimize underestimation, calibration using 4-*O-*Me-GlcA standards is recommended for quantification whenever possible. For hardwood samples analyzed in “Comparison of uronic acid quantification in various samples”, UA concentrations were therefore determined using both GlcA- and 4-*O-*Me-GlcA-based calibrations for comparison (see “Comparison of uronic acid quantification in various samples”).

#### Influence of aldobiuronic acid-type linkages

##### Isolation and structural characterization of glucuronoxylan oligosaccharides

Aldobiuronic acid-type linkages, representing glycosidic bonds between a neutral sugar and a UA, are known for their exceptional hydrolytic stability [33]. To evaluate their influence on UA quantification, GXOS containing 4-*O-*Me-GlcA substituents were isolated from a beechwood xylan standard. Enzymatic depolymerization was conducted using two *endo*-xylanases, belonging to glycoside hydrolase families GH10 and GH11, followed by comparative evaluation of product spectra and yields via HPAEC-PAD and MS.

**Fig. 8:**
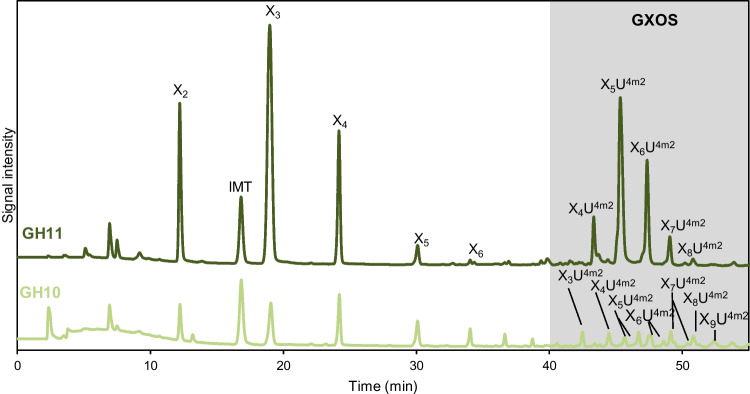
HPAEC chromatogram detected by PAD of the (optimized) enzymatic digestion using glycoside hydrolases (GH) 10 and 11. Isomaltotriose (IMT) was added as an internal standard. The nomenclature of the detected xylooligosaccharides and glucuronoxylan oligosaccharides (GXOS) follows Fauré, Courtin [36] (see text)

HPAEC-PAD chromatograms (Fig. 8) revealed different product distributions: GH10 xylanase generated a broad range of shorter oligosaccharides (DP 3–9), whereas GH11 xylanase predominantly produced fragments with a greater DP (DP 4–8). According to literature, the GH10 enzyme preferentially cleaves unsubstituted xylose sequences, yielding mainly xylobiose and substituted GXOS carrying 4-*O-*Me-GlcA at the non-reducing end [34, 35]. In contrast, GH11 requires three consecutive unsubstituted xylose units and typically produces XU^4m2^XX as the smallest GXOS [34]. Owing to its simpler product spectrum and higher specific activity, GH11 xylanase digestion (0.25 U/mg substrate, 50 °C, 5 h, chromatogram Fig. 8) was selected for preparative isolation of GXOS. The nomenclature of the obtained oligosaccharides follows the systematic convention proposed by Fauré, Courtin [36], in which X denotes unsubstituted xylose residues and U indicates substitution by a UA (position in the oligosaccharide remains unclear). Superscripts and subscripts specify the position and type of modification (e.g., X_3_U^4m2^ for an 4*-O*-methylated GlcA attached at *O*2 of a xylose in a backbone with three xylose units).

**Fig. 9:**
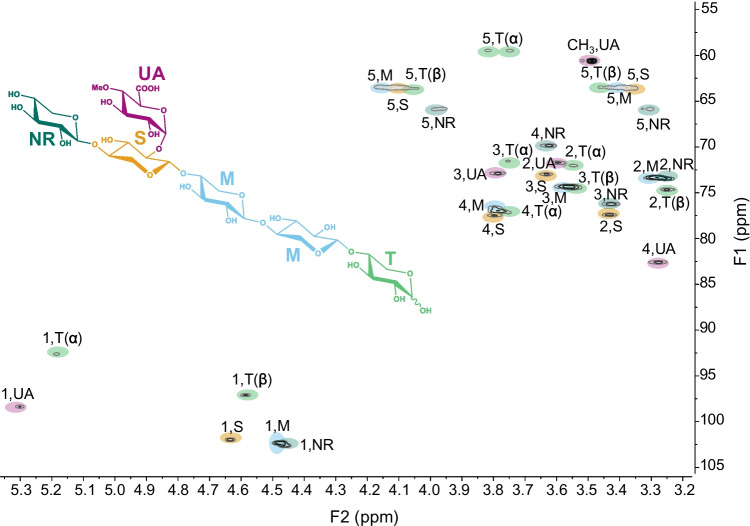
HSQC spectrum of the isolated XU^4m2^XXX standard (in D_2_O, referenced against acetone *δ*_*H*_ = 2.22 ppm/*δ*_*C*_ = 30.89 ppm). Signal nomenclature corresponds to the position of the assigned *C*/*H* signal within the monosaccharide and the respective monomer unit of the structure shown

Neutral XOS were removed by anion exchange SPE according to Miguez, Fernandez-Polo [37] and the remaining GXOS mixture (X_4 - 8_U^4m2^, Fig. 9, GH11) was separated semi-preparatively by HILIC. Despite extensive chromatographic optimization, individual fractions exhibited purities between 83 and 86% due to partial co-elution of adjacent DP species. Structural elucidation of the major component (X_5_U^4m2^) was achieved by 2D-NMR spectroscopy (see Table S3 for chemical shifts in supplementary data).

The ^1^H-NMR spectrum (SI Fig. S4) showed characteristic resonances for the methyl group of the UA (*δ*_*H*_ = 3.48 ppm) and the anomeric protons (*δ*_*H*_ = 5.29–4.45 ppm). Cross peaks in the HSQC (Fig. 9) and HSQC-TOCSY (SI Fig. S5) spectra enabled assignment of spin systems corresponding to terminal, substituted, unsubstituted, and reducing xylose units, as well as the UA unit. H2BC experiments (SI Fig. S6) allowed for the identification of *C1–C5* of each unit.

The HMBC experiment (SI, Fig. S7) provided long-range ^3^*J*_*C*,*H*_ correlations that identified inter-residue linkages and verified the *O*4-methylation of the GlcA residue. A ^3^*J*_*C*,*H*_ cross-peak between the anomeric proton of the non-reducing xylose unit (*δ*_*H*__1_ = 4.45 ppm) and the C4 resonance of the substituted residue (*δ*_*C*__4_ = 77.5 ppm) indicates that the 4-*O-*Me-GlcA substituent is located on the second xylose unit from the non-reducing end, consistent with the structure XU^4m2^XXX. A direct HMBC cross-peak that would unambiguously prove linkage of the 4-*O-*Me-GlcA to the *O*2 position of this xylose was not observed. However, the *C*2/*H*2 cross-peak of the substituted xylose in the HSQC spectrum is markedly downfield shifted relative to the other *C*2 signals, consistent with *O*2 substitution.

For GXOS with a greater DP, the substitution pattern could not be fully resolved, among others, due to limited sample quantities, but based on enzymatic cleavage characteristics, analogous substitution was assumed.

##### Quantification of uronic acids in glucuronoxylan oligosaccharides by LC–MS

To evaluate the influence of the aldobiuronic acid linkage and the DP of the xylan backbone on UA quantification, three GXOS of sufficient purity (> 80%) were analyzed using the optimized LC–MS method. Each sample solution contained an equivalent molar amount of 4-*O-*Me-GlcA, and recoveries were referenced against the corresponding 4-*O-*Me-GlcA standard. The results are summarized in Table 5.

**Table 5:** Recoveries of 4-O-methyl-glucuronic acid (as total uronic acid) within the isolated glucuronoxylan oligosaccharides

Structure	Recovery (% monosaccharide)
XU^4m2^XXX (X_5_U^4m2^)	73.93 ± 1.86
X_6_U^4m2^	73.09 ± 0.47
X_7_U^4m2^	64.33 ± 0.24

The data clearly indicate that UAs that are covalently bound through the aldobiuronic acid linkage were recovered at substantially lower levels (up to 35% below the monosaccharide reference) suggesting incomplete cleavage and a higher hydrolytic stability of these linkages. The recoveries obtained for XU^4m2^XXX and X₆U^4m2^ were comparable, indicating that the xylan chain length has no distinct effect. In contrast, X₇U^4m2^ exhibited approximately 9% lower recovery, possibly reflecting altered release kinetics of the substituted residue. However, as the precise substitution site could only be fully confirmed for XU^4m2^XXX, it cannot be ruled out that a different position of the 4-*O-*Me-GlcA substitution, rather than the number of xylose units, accounts for the observed difference.

The transferability of these findings to native polysaccharides remains uncertain, yet a systematic underestimation of the UA content can be expected for materials containing stable aldobiuronic acid linkages. Modifying the reaction time during acid degradation, either by extending or shortening incubation, did not yield a consistent improvement in recovery.

Another potential uncertainty arises from the sulfuric acid pretreatment used for GX-containing wood samples. Partial hydrolysis of aldobiuronic acid linkages during this step cannot be excluded, which might particularly affect soluble materials, whereas the impact on hardwood residues is likely less pronounced.

For a more comprehensive evaluation, future studies should extend the present analysis to rhamnogalacturonan I-derived oligosaccharides and hydrocolloid structures that, different from wood samples, do not require a pre-hydrolysis and thus commonly retain intact aldobiuronic acid linkages to be cleaved in the procedure applied to form 5FFA. Reliable quantification of such UA-containing materials may require either optimization of the parameters required to form 5FFA or the application of correction factors based on calibration with UA-containing oligosaccharide standards. However, this approach is currently limited by the restricted commercial availability of well-defined reference compounds.

### Comparison of uronic acid quantification in various samples

The isolated fiber materials and other milled samples were analyzed for their total UA contents using the optimized LC–MS-based method as well as the spectrophotometric assay according to Blumenkrantz and Asboe-Hansen [14] (Table 6). The comparison reveals notable discrepancies between the two methods without a clear trend: in some cases, such as karaya gum and brown algae, the spectrophotometric method yields significantly higher UA contents, whereas for gum arabic the results largely agree, and in other samples the spectrophotometric method reports lower values.

**Table 6:** Comparison of the total uronic acid (UA) contents of plant and algae materials (*IF*, insoluble fiber; *SF*, soluble fiber) analyzed by the LC–MS method applying the optimized parameters according to Table 1, and the spectrophotometric method according to Blumenkrantz and Asboe-Hansen [14] (*RSD*, percent relative standard deviation (intra-day), n = 3). The calibrations were performed using the UA that was found dominant in the analysis of the monosaccharide composition (see SI Figs. S1–S3)

		**LC–MS**		**Spectrophotometry**^**e**^	
		Total UA (mg/100 mg)	RSD (%)	Total UA (mg/100 mg)	RSD (%)
**Karaya gum**		23.87 ± 0.52^a^	2.18	34.79 ± 4.72	13.57
**Xanthan**		23.14 ± 0.37^b^	1.62	17.19 ± 0.38	2.24
**Gum arabic**		15.41 ± 0.65^b^	4.21	14.35 ± 0.64	4.49
**Beechwood**		2.13 ± 0.09^b^	4.23	1.72 ± 0.11	6.29
		2.28 ± 0.02^c^	0.88	1.70 ± 0.12	7.06
**Eucalyptus wood**		3.82 ± 0.07^b^	1.83	3.09 ± 0.02	0.65
		4.14 ± 0.07^c^	1.70	2.91 ± 0.02	0.69
**Brown seaweed**	**SF**	39.05 ± 1.46^d^	3.74	57.21 ± 11.25	19.66
**Brown seaweed**	**IF**	79.97 ± 0.30^d^	0.38	114.77 ± 0.78	0.68

^a^analyzed with the optimized conditions for GalA + GlcA ^b^analyzed with the optimized conditions for GlcA ^c^analyzed with the optimized conditions for GlcA, but using the 4-O-Me-GlcA calibration ^d^analyzed with the optimized conditions for ManA + GulA ^e^analyzed by using the conditions according to "Colorimetric assay"

These findings must be interpreted considering the previously presented validation data (“Method validation”), which suggested a significant overestimation of UA by the spectrophotometric method in brown algae. Values exceeding 100% in the IF sample further question the reliability of the spectrophotometric quantification. Hardwood samples were quantified using both GlcA and 4-*O-*Me-GlcA calibrations; the latter yielded slightly higher values, although the results were largely comparable.

A systematic underestimation is expected for hydrocolloids and hardwoods, both of which contain aldobiuronic acid linkages, consistent with the results from “Quantification of uronic acids in glucuronoxylan oligosaccharides by LC–MS”. This underestimation affects both methods equally, as incomplete cleavage of aldobiuronic acid linkages during concentrated sulfuric acid hydrolysis remains a limiting factor that cannot be compensated for by calibration or internal standards.

Inter-day precision is given in Table 7 for selected sample materials. For the LC–MS method, values were below 2.2% for all three sample materials, demonstrating excellent reproducibility across measurement days. In contrast, the spectrophotometric method showed substantially higher inter-day variation, with values of 15.42% (karaya gum), 53.10% (xanthan), and 18.31% (brown seaweed SF). One-way ANOVA (α = 0.05) confirmed that no significant differences existed between the two measurement days for any of the sample materials analyzed by LC–MS (karaya gum: p = 0.620; xanthan: p = 0.254; brown seaweed SF: p = 0.191). For the spectrophotometric method, significant inter-day differences were detected for xanthan (p = 0.001) and brown seaweed SF (p = 0.001), while karaya gum did not reach statistical significance (p = 0.101), though its CV already indicates poor reproducibility. These results further underline the superior inter-day robustness of the LC–MS method compared to the spectrophotometric approach.

**Table 7:** Inter-day precision of total uronic acid determination analyzed by LC–MS method applying the optimized parameters according to Table 1, and the spectrophotometric method according to Blumenkrantz and Asboe-Hansen [14] for selected sample materials (*SF*, soluble fiber, n = 4, days = 2)

		**LC–MS**	**Spectrophotometry**^**d**^
		Inter-day precision (%)	Inter-day precision (%)
**Karaya gum**		2.16^a^	15.42
**Xanthan**		1.22^b^	53.10
**Brown seaweed**	**SF**	1.44^c^	18.31

^a^Analyzed with the optimized conditions for GalA + GlcA

^b^Analyzed with the optimized conditions for GlcA

^c^Analyzed with the optimized conditions for ManA + GulA

^d^Analyzed by using the conditions according to “Colorimetric assay”

Additionally, bubble tea pearls, containing alginate-derived ManA and GulA as well as GlcA containing xanthan, were analyzed. Only trace amounts of GlcA were detected within the monosaccharide composition of the bubble tea pearls, indicating a low GlcA content. Testing the total UA (ManA + GulA parameters, Table) in alginate standards spiked with varying xanthan concentrations showed minimal effects of xanthan addition (Table 8). Due to the low degradation rate of the GlcA moiety in xanthan at 60 °C, the analysis in bubble tea samples effectively quantifies only ManA + GulA. While this limits the LC–MS method’s capacity for total UA determination, it offers opportunities for selective quantification of specific UA fractions. Further studies are needed to define the threshold at which other UA interfere with selective ManA + GulA quantification.

**Table 8:** Uronic acid content quantified as mannuronic acid and guluronic acid in an alginate standard spiked with varying amounts of xanthan, as well as in bubble tea material

	**Uronic acid (mg/100 mg)**
Alginate	101.87 ± 2.35
Alginate + 2% xanthan	104.26 ± 0.16
Alginate + 10% xanthan	103.22 ± 0.64
Alginate + 50% xanthan	104.46 ± 1.50
Bubble tea	33.26 ± 1.47

## Conclusion

In conclusion, this study advances the state of the art by overcoming the long-standing limitations of conventional colorimetric UA assays, such as matrix-induced interferences and poor reproducibility. Moving beyond previous specialized applications that focused primarily on GalA, we demonstrate that the optimized LC–MS stable isotope dilution approach serves as a versatile and superior tool for the accurate assessment of total UA contents across a wide range of polysaccharides.

By optimizing the heating time and temperature during the acid degradation step, GlcA, ManA, and GulA were successfully integrated into the analysis. The process was tailored to the specific UA composition of the samples, ensuring improved and consistent sensitivity for the different UAs and UA pairs. Furthermore, the systematic evaluation of structural influences, such as the DP and 4-*O*-methylation, provides new insights into the method’s robustness. Under these optimized conditions, the DP of polyuronic acids was found to have no measurable effect on quantification accuracy. In contrast, UAs occurring in aldobiuronic acid linkages, such as in isolated GXOS, were underestimated, highlighting the need for further method development to improve cleavage efficiency and recovery in such structures. Overall, the developed LC–MS method represents a significant methodological progression, ensuring reliable quantification of total UA content in complex matrices such as alginates and plant exudates.

## Supplementary Information

Below is the link to the electronic supplementary material.

Supplementary file1 (DOCX 20.9 MB)

## Data Availability

Additional data are made available as supplementary materials and/or will be made available upon request.
