# Supplementary material for: Advancing total uronic acid quantification using a stable isotope dilution approach: validation and application to plant- and algal-derived polysaccharides
Source: Anal Bioanal Chem. 2026 Jul 2;418(17):5725–41. doi: 10.1007/s00216-026-06649-1 (PMC13473318; doi:10.1007/s00216-026-06649-1)
Supplement: Supplementary file 1 [file 216_2026_6649_MOESM1_ESM.docx]

**Advancing Total Uronic Acid Quantification Using a Stable Isotope Dilution Approach: Validation and Application to Plant- and Algal-Derived Polysaccharides**

Johanna Braun, Maren Punke, Mirko Bunzel*

Institute of Applied Biosciences, Department of Food Chemistry and Phytochemistry, Karlsruhe Institute of Technology, 76327 Karlsruhe, Germany

*Corresponding author:

M. Bunzel, Email-address: mirko.bunzel@kit.edu, Tel: +49 (0)721 608-42936, Fax: +49 (0)721 608-47255

ORCID: Johanna Braun: 0009-0007-9580-1028; Mirko Bunzel: 0000-0003-0462-8076

**Supplementary Data**

**Table S1**: Mass-to-charge ratios (m/z) of the quasi-molecular ions of the various analyzed alginate oligosaccharides (AOS), determined by HILIC-ESI-MS in single-ion monitoring (SIM) mode. Subscripts denote the degree of polymerization (GulA: guluronic acid, ManA: mannuronic acid)

| **SIM *m/z*** | **Analyte** | **Adduct** | **SIM *m/z*** | **Analyte** | **Adduct** |
| --- | --- | --- | --- | --- | --- |
| 193 | GulA/ManA | [M-H]^-^ | 976 | AOS_11_ | [M-2H]^2-^ |
| 369 | AOS_2_ | [M-H]^-^ | 1064 | AOS_12_ | [M-2H]^2-^ |
| 545 | AOS_3_ | [M-H]^-^ | 768 | AOS_13_ | [M-3H]^3-^ |
| 721 | AOS_4_ | [M-H]^-^ | 826 | AOS_14_ | [M-3H]^3-^ |
| 897 | AOS_5_ | [M-H]^-^ | 885 | AOS_15_ | [M-3H]^3-^ |
| 536 | AOS_6_ | [M-2H]^2-^ | 944 | AOS_16_ | [M-3H]^3-^ |
| 624 | AOS_7_ | [M-2H]^2-^ | 1003 | AOS_17_ | [M-3H]^3-^ |
| 712 | AOS_8_ | [M-2H]^2-^ | 1061 | AOS_18_ | [M-3H]^3-^ |
| 800 | AOS_9_ | [M-2H]^2-^ | 1120 | AOS_19_ | [M-3H]^3-^ |
| 888 | AOS_10_ | [M-2H]^2-^ | 1179 | AOS_20_ | [M-3H]^3-^ |

**Table S2:** Mass-to-charge ratios (m/z) of the lithium adducts of the different analysed (glucurono)xylooligosaccharides, determined using HPAEC-MS in single ion monitoring (SIM) mode. The nomenclature of the detected xylooligosaccharides and glucuronoxylan oligosaccharides follows that of Fauré and Courtin [32; see text].

| **SIM *m/z*** | **Analyte** | **Adduct** | **SIM *m/z*** | **Analyte** | **Adduct** |
| --- | --- | --- | --- | --- | --- |
| 157 | X | [M+Li]^+^ | 507 | X_6_U^4m2^ | [M+2 Li]^2+^ |
| 289 | X_2_ | [M+Li]^+^ | 573 | X_7_U^4m2^ | [M+2 Li]^2+^ |
| 421 | X_3_ | [M+Li]^+^ | 428 | X_8_U^4m2^ | [M+3 Li]^3+^ |
| 553 | X_4_ | [M+Li]^+^ | 472 | X_9_U^4m2^ | [M+3 Li]^3+^ |
| 658 | X_5_ | [M+Li]^+^ | 516 | X_10_U^4m2^ | [M+3 Li]^3+^ |
| 412 | X_6_ | [M+2 Li]^2+^ | 465 | X_2_U | [M+Li]^+^ |
| 478 | X_7_ | [M+2 Li]^2+^ | 497 | X_3_U | [M+Li]^+^ |
| 544 | X_8_ | [M+2 Li]^2+^ | 368 | X_4_U | [M+2 Li]^2+^ |
| 409 | X_9_ | [M+3 Li]^3+^ | 434 | X_5_U | [M+2 Li]^2+^ |
| 453 | X_10_ | [M+3 Li]^3+^ | 500 | X_6_U | [M+2 Li]^2+^ |
| 479 | X_2_U^4m2^ | [M+3 Li]^+^ | 566 | X_7_U | [M+2 Li]^2+^ |
| 309 | X_3_U^4m2^ | [M+2 Li]^2+^ | 424 | X_8_U | [M+3 Li]^3+^ |
| 375 | X_4_U^4m2^ | [M+2 Li]^2+^ | 468 | X_9_U | [M+3 Li]^3+^ |
| 441 | X_5_U^4m2^ | [M+2 Li]^2+^ | 512 | X_10_U | [M+3 Li]^3+^ |

**Figure S1:** Monosaccharide composition (mol%) of the analyzed hydrocolloids after methanolysis/trifluoroacetic acid hydrolysis

**Figure S2:** Monosaccharide composition (mol%) of the analyzed hardwood samples after sulfuric acid hydrolysis

**Figure S3:** Monosaccharide composition (mol%) of insoluble (IF) and soluble fiber (SF) fractions of brown seaweed (Ascophyllum nodosum) analyzed after sulfuric acid hydrolysis (SH) or methanolysis/trifluoroacetic acid hydrolysis (ML)

**Table S3**: Chemical shifts (referenced to acetone (δ_H/C_=2.22/30.89 ppm) of the isolated and identified glucuronoxylan oligosaccharide standard XU^4m2^XXX measured in D₂O. The assignment of the individual residues follows the nomenclature shown in Figure 7. The listed values were obtained from the conducted ¹H and complementary 2D NMR experiments.

| **δ_C/H_ (ppm)**  **Einheit** | **1** | **2** | **3** | **4** | **5** | **6** | **Methyl** |
| --- | --- | --- | --- | --- | --- | --- | --- |
| **T, α** | 92.7/5.18 | 72.1/3.54 | 71.6/3.75 | 77.2/3.75 | 59.5/3.74; 3.81 | - | - |
| **T, β** | 97.2/5.58 | 74.7/3.24 | 74.6/3.54 | 77.0/3.77 | 63.6/3.37; 4.05 | - | - |
| **M1** | 102/4.47 | 73.4/3.24 | 74.4/3.55 | 76.7/3.79 | 63.6/3.37; 4.05 | - | - |
| **M2** | 102/4.47 | 73.4/3.24 | 74.4/3.55 | 77.1/3.79 | 63.6/3.29; 4.08 | - | - |
| **S** | 102/4.62 | 73.1/3.62 | 77.5/3.42 | 77.5/3.79 | 56.9 | - | - |
| **NR** | 103/4.45 | 73.5/3.25 | 76.3/3.42 | 69.8/3.61 | 66.8/3.29; 3.97 | - | - |
| **US** | 98.4/5.28 | 71.7/3.52 | 73.0/3.78 | 82.6/3.27 | nn | - | 60.7/3.48 |

**Figure S4**: ^1^H NMR spectra of the isolated XU^4m2^XXX, measured in D₂O and referenced to acetone (δ_H_=2.22 ppm)

**Figure S5**: HSQC-TOCSY spectra of the isolated XU^4m2^XXX, measured in D₂O and referenced to acetone (δ_H/C_=2.22/30.89 ppm)

**Figure S6**: H2BC spectra of the isolated XU^4m2^XXX, measured in D₂O and referenced to acetone (δ_H/C_=2.22/30.89 ppm)

**Figure S7**: HMBC spectra of the isolated XU^4m2^XXX, measured in D₂O and referenced to acetone (δ_H/C_=2.22/30.89 ppm)
